# Parental Perceptions and Decisions Regarding Maintaining Bilingualism in Autism

**DOI:** 10.1007/s10803-020-04528-x

**Published:** 2020-05-09

**Authors:** Katie Howard, Jenny Gibson, Napoleon Katsos

**Affiliations:** 1 Theoretical and Applied Linguistics, University of Cambridge, Cambridge, UK; 2 Faculty of Education, University of Cambridge, Cambridge, UK; 3 Jesus College, Jesus Lane, Cambridge, CB5 8BL UK

**Keywords:** Bilingualism, Autism, Parental experiences

## Abstract

A growing body of evidence suggests that bilingual exposure does not negatively impact children on the autism spectrum. This study sought to illuminate parents’ perceptions and choices regarding maintaining bilingualism in autism. Semi-structured interviews were conducted with 16 family members in England and Wales. Data were analysed using interpretative phenomenological analysis (IPA). Although parents expressed positive attitudes towards bilingualism, these views were not always congruent with their language practices. Instead, several factors influenced decisions about language maintenance in autism, including the severity of the child’s autism, advice received, and the importance of English as the dominant societal language. This article calls for greater support for families in making language decisions that are suitable for the individual child and their family.

## Background

Common misconceptions about bilingualism can complicate families’ decisions about whether to speak one or more language(s) to their children. Such decisions are made more complex when a child is diagnosed with a developmental disorder. The notions that only typically-developing children can be bilingual or that bilingualism somehow causes developmental disorders are deeply misleading (Baker 2011; Genesee et al. 2004). In fact, there is a growing body of evidence suggesting that multilingual exposure does not have a negative impact on children with developmental disorders, and could potentially have positive effects for their social and linguistic development (Dai et al. 2018; Drysdale et al. 2015; Uljarević et al. 2016).

Autism Spectrum Disorder is a developmental condition characterised by ‘persistent deficits in social communication and social interaction across multiple contexts’ and ‘restricted, repetitive patterns of behaviour’ (APA 2013). Communication and language difficulties often consist of challenges in pragmatics (Naigles and Chin 2015), echolalia or delayed speech (Baron-Cohen 2008), and, in around 25% of cases, no verbalisation (Tager-Flusberg et al. 2005). Investigating a range of stakeholders’ perspectives on the terminology used to describe autism, Kenny et al. (2016) found that ‘on the autism spectrum’ was widely accepted and this term will therefore be used in the current study.^1^

In the UK, autism affects over 700,000 people and their families (McCall 2017), while prevalence estimates suggest that approximately 1.5% of 5–9 year-olds may have an autism spectrum condition (Baron-Cohen et al. 2009). With a rising number of bilingual children in UK schools, the interaction between autism and bilingualism is an increasingly common experience. Rather than using the term ‘English as an additional language’, which is most frequently used in educational settings in England, we refer to children in this study as ‘bilingual’, that is, as those who ‘need and use two or more languages (or dialects) in their everyday lives’ (Grosjean 2010, p. 4). Our reasons for this terminological preference are threefold: our sample includes native English speakers in Wales who are educated through the medium of Welsh (who would not be classified as EAL); many of the children were simultaneous bilinguals and therefore English was not an ‘additional’ language for them; and the literature on autism and bilingualism routinely adopts the terms ‘bilingual’ and ‘multilingual’. We distinguish between ‘bilingual’ for descriptions of individuals’ linguistic profiles and ‘multilingual’ to describe the interaction of multiple languages within socio-geographic spaces (Wei 2014), e.g. multilingual families, environments or approaches.

### Autism and Bilingualism

An emerging body of research documents the interaction between autism and bilingualism, focusing primarily on the decisions that multilingual families make about language use if their child is diagnosed with autism. These studies consistently find that parents are advised to adopt a monolingual approach (Hampton et al. 2017; Yu 2013). This is contrary to the growing evidence that children on the autism spectrum have the capacity to learn or maintain a second language (Peterson et al. 2012; Valicenti-McDermott et al. 2013). Indeed, no research to date suggests that bilingual maintenance is detrimental to the social, cognitive and linguistic development of children on the autism spectrum (Drysdale et al. 2015; Uljarević et al. 2016). Instead, studies have found that bilingual children on the autism spectrum perform similarly to their monolingual counterparts with regards to expressive and receptive vocabulary and language (Dai et al. 2018; Hambly and Fombonne 2012), pragmatic abilities (Reetzke et al. 2015) and cognitive functioning (Valicenti-McDermott et al. 2013).

### Parental Language Choices

Parents of bilingual children on the autism spectrum are faced not only with the challenge of language choices, but also with differing advice from practitioners about the impact of language selection on a child’s development. Existing studies found that parents are often discouraged by practitioners (both educational and health professionals) to maintain their heritage language (Baker 2013; Yu 2009, 2016). This advice to adopt a single-language approach may be given to parents with the aim of mitigating potential language delays (Bird et al. 2016b; Ijalba 2016; Jegatheesan 2011).

Nevertheless, the recommendation to adopt a monolingual approach is ‘highly problematic’ (Yu 2013, p. 11). Suggesting monolingualism can, in many cases, ‘isolate children from social and cultural activity in the community’ (Baker 2011, p. 345), and restrict their social interaction with family members (Bird et al. 2016b). Further, asking parents to use their non-native language may be unhelpful if they lack fluency and are inadvertently modelling its use in an incorrect manner (Drysdale et al. 2015). Yu argues that it is unrealistic to expect parents to reduce, or worse still abandon, the use of their own native language, suggesting that such advice is not only untenable, but ‘at odds with their ways of life’ (Yu 2016, p. 425).

Despite the frequent advice to provide a monolingual environment, parents often consider bilingualism to be a valuable asset and are eager for their children to be exposed to their heritage language as well as the dominant societal language, which, in much of the existing research, is English. Certain studies outline the myriad benefits of bilingual exposure for children on the autism spectrum. These include: developing multicultural identities and the preservation of heritage (Jegatheesan 2011; Yu 2013); participation in religious life (Jegatheesan 2011); enriched relationships with, and access to, immediate and extended family members (Bird et al. 2016b; Hampton et al. 2017; Yu 2013); and cognitive skills related to attention (Gonzalez-Barrero and Nadig 2019).

### Recommendations

Existing studies provide recommendations to counter the well-intentioned but misleading advice that ‘one language is best’ (Lim et al. 2018; Paradis and Govindarajan 2018). To ensure bilingualism is a viable possibility, children on the autism spectrum may require more opportunities to hear and use their home language, especially those who receive more exposure to the dominant language (Paradis and Govindarajan 2018). Accordingly, providing speech and language interventions in both languages may help to alleviate the common risk of language attrition in the minority language (Bird et al. 2016a). Considerations about cultural factors must also come to the fore when practitioners are advising multilingual parents, as certain communities may have concerns about the stigmatization of an autism diagnosis (Fox et al. 2017; Ijalba 2016).

Beauchamp and MacLeod (2017) and Lim et al. (2018) call for more support for families wishing to raise their child on the autism spectrum bilingually. Such support could be possible through increased dialogue between families and practitioners, with particular attention given to parents’ existing language practices (Uljarević et al. 2016). Family-school partnerships are particularly important for this group, because parents of bilingual children are often under-represented in school structures (Arnot et al. 2014) while parents of children on the autism spectrum report that they are less satisfied with the quality and quantity of school communication than parents of typically-developing children (Zablotsky et al. 2012). By understanding the perceptions and choices of parents, and the challenges that discourage them from choosing a multilingual approach, researchers and practitioners will be better placed to provide evidence-based recommendations. This is particularly important given that parental perceptions often inform their decision-making processes [e.g. as has been found in the domains of interventions (Hebert 2014), and family planning (Selkirk et al. 2009)].

### Context of the Current Study

The UK provides an interesting case study for the increasingly common interaction between autism and bilingualism, particularly given linguistic differences between its various jurisdictions. The current study is concerned with parental perceptions and choices of maintaining bilingualism in autism in two linguistically different settings: England and Wales. In England, children with English as an Additional language (EAL) represent more than one in five (21.2%) primary school pupils (DfE 2018). Despite increasingly multilingual classrooms in England, the education system is almost entirely monolingual. The linguistic landscape in Wales is very different. Fewer children speak EAL in Wales than in England, although this number is rising to around 7% of pupils (WG 2015). However, around a quarter of school-aged children in Wales attend a Welsh-medium school (WG 2018). Welsh-medium education is selected by parents for cultural, educational and employment reasons (Hodges 2011; O’Hanlon 2014). Under the auspices of the Welsh Language Strategy, the Welsh Government are aiming to reach one million Welsh speakers in Wales by 2050 (WG 2017). By taking this comparative approach between countries that are geographically close but linguistically different, we aim to promote international and intercultural dialogue within the growing field of bilingualism and autism.

## Research Questions

The aim of the current study was to elucidate what Baker describes as the ‘texture and nuance that exists in the lives of all multilingual families of children with autism’ (2013, p. 527). By drawing on the micro-perspective of individual families’ language practices, and the macro-perspective of the language contexts in which they live, the current study was informed by the following research questions:

1. What are parents’ perceptions about the value of bilingualism when raising a child on the autism spectrum?
2. To what extent do these perceptions influence their language choices?
3. What are the consequences of choosing a more monolingual or multilingual approach?
4. How do perceptions and language choices differ between parents who are raising their children in a country where the education system is predominantly monolingual (England) and parents raising children in a country where education promotes bilingualism (Wales)?

## Methods

### Interpretative Phenomenological Analysis

In the current study, interpretative phenomenological analysis was considered a useful methodological approach given its commitment to understanding the lived experience of participants (Smith et al. 2009) and its increasing use in autism research (Howard et al. 2019). IPA is characterised by a ‘double hermeneutic’ (Smith et al. 2009) whereby both the researcher and the participant are seeking to interpret the experiences described. However, we argue that in this study a ‘triple hermeneutic’ may be at play in which the researcher and participant are not only interpreting the participant’s own experiences but are seeking to understand the sense-making of their children with regard to their linguistic identity and proficiency.

### Ethical Considerations

Ethical approval was sought and granted from the School of the Humanities and Social Sciences at the University of Cambridge before the study began (Case No: 17/136). Participants were provided with an information sheet detailing the aims and procedures of the research and informed consent was obtained from all individual participants included in the study. Audio recordings were safely stored in a password-protected file. All participants were given pseudonyms to protect their anonymity.

### Participants

Participants (n = 16) were purposively recruited through contact with schools in England and Wales and through social media posts. Parents were included in the current study if their child: (1) had been diagnosed with autism in the UK, and (2) was exposed to more than one language on a daily basis. The majority of participants (n = 14) were mothers of a bilingual child on the autism spectrum. One bilingual father and one monolingual grandmother were also interviewed together with the two respective mothers. One bilingual mother (Dasia) had a son and a daughter on the autism spectrum and spoke about her experiences parenting both children, who were adults aged 18 and 21 years-old at the time of interview. All other participants' children were aged between 6 and 12 years-old at the time of interview. A wide range of languages were spoken by participants and/or their families; some parents were native English speakers whose partner spoke a different language, while others were either first- or second-generation migrants. To ensure that participants are unidentifiable, the languages spoken by participants or their children have not been linked to individual participants. These languages were: Arabic, Bengali, French, Gujarati, Hindi, Italian, Polish, Punjabi, Spanish, Turkish, Urdu and Welsh.

### Procedures

Semi-structured, phenomenological interviews were conducted to draw out the experiences of parenting a bilingual child on the autism spectrum. The first author conducted all the interviews for the sake of continuity, drawing on previous experience and training in qualitative research and professional experience of working with parents of children with additional languages and additional learning needs. Described by Gubrium, Holstein, and Marvasti as ‘a virtual window on experience’ (Gubrium et al. 2012, p. 30), semi-structured interviews are an appropriate research instrument for phenomenologists because they provide descriptions of participants’ daily lives, and generate detailed information about their interpretations and perceptions of such experiences. An interview schedule with open-ended questions about parents’ experiences of raising a child on the autism spectrum and making decisions about language use was devised, reviewed and piloted for feasibilty. Participants were given an outline of the discussion topics prior to the interview. Most participants were willing to be interviewed in English, however two participants opted to have an interpreter present as they felt more comfortable expressing their views in their native language. Interviews lasted between 13 and 46 min (average = 28 min) and took place in participants’ homes (n = 7), their children’s schools (n = 6) or another location of their choice (n = 1). Demographic information and interview details are provided in Table 1.

**Table 1:** Demographic information and interview details

Participant	Relation to child	Gender of child	Interview location	Interpreter used	Interview length	Country of residence	Multi- or mono- approach
Anna^a^	Mother	M	School	No	35.32	Wales	Multi
Baheela	Mother	F	School	Yes	12.58	England	Multi
Chandra	Mother	M	School	No	28.10	England	Multi
Dasia	Mother	1M & 1F	Conference centre	No	23.13	England	Mono
Davesh^b^	Father	M	Home	No	35.17	England	Mono
Eleanora	Mother	M	School	No	26.59	England	Multi
Hira	Mother	M	Home	No	35.17	England	Mono
Julie	Mother	M	Home	No	26.28	Wales	Multi
Katherine	Mother	M	Home	No	22.23	Wales	Multi
Lena	Mother	M	Home	Yes	16.21	England	Multi
Magdalena	Mother	M	Home	No	27.43	England	Mono
Mary	Grandmother	M	School	No	35.32	Wales	Multi
Molly	Mother	M	School	No	30.50	Wales	Mono
Nabani	Mother	M	School	No	42.37	England	Mono
Roberta	Mother	M	Home	No	46.04	England	Multi
Roshan	Mother	F	Home	No	18.53	England	Mono

^a^Interviewed with Mary

^b^Interviewed with Hira

### Data Analysis

Each interview was transcribed verbatim within 3 h of the interview to ensure that the memory of the interaction was fresh and detailed notes were taken with first impressions, contextual factors and key themes (Magnusson and Marecek 2015). Given the focus on content over particular linguistic features, a denaturalised approach to transcription was adopted whereby the meanings of speech took precedence over form (Oliver et al. 2005). Following transcription, interview data were analysed by the first author, who had undergone training on using interpretative phenomenological analysis. Analyses followed the IPA guidelines provided by Smith et al. (2009). The first transcript was read and re-read with descriptive comments made in the left margin and emergent themes noted in the right margin. The same process was conducted for each transcript and a list of superordinate themes and subthemes was compiled along with their corresponding verbatim quotations. Themes were reviewed and discussed between all authors until a consensus was reached.

### Rigour

Issues surrounding the validity of qualitative studies with interviews include the trustworthiness of participants’ reports and the quality and depth of the interviewing (Kvale 1995) along with a ‘sensitivity to context’ (Yardley 2000). As such, a number of strategies were employed to increase methodological rigour in this study. First, the first author carried out all the interviews for the sake of continuity. Data were transcribed directly after each interview for the sake of accuracy, and detailed field notes taken to ensure a thorough coverage of the participants’ experiences. Following the analysis of each transcript, participants were contacted and provided with a summary of themes. This process of member-checking enabled participants to verify the interpretations of the data and gave them an opportunity to clarify any ambiguities or misrepresentations (Lincoln and Guba 1985). The interpretation of interview data and the list of superordinate themes were compiled in collaboration with the research team and were reviewed by an independent researcher to increase the confirmability of the findings.

## Results

Eight families indicated that they had opted for a more multilingual approach to raising their child on the autism spectrum, while six families reported opting for a more monolingual approach (i.e. using mainly English). Three out of the four families interviewed in Wales opted to maintain Welsh, while five out of ten families in England opted to maintain the home language. Three broad themes were extracted from the data, which are presented in Table 2, along with their sub-themes. The superordinate themes were: (a) parental perceptions about the value of bilingualism, (b) factors influencing language decisions, (c) consequences of language choices.

**Table 2:** Themes

Superordinate theme	Sub-theme
A. Parental perceptions about the value of bilingualism	(1) Impact on communication (2) Cultural value (3) Impact on cognition
B. Factors influencing language decisions	(1) Family (2) Advice received (3) Feasibility of bilingualism (4) Practical considerations (5) The role of English
C. Consequences of language choices	(1) Changes over time (2) Family well-being (3) Education

Each of these three superordinate themes and their concomitant sub-themes will be considered from the perspectives of families in two distinct groups outlined in Table 3: parents who opted for a more multilingual approach (group 1) and those who chose a more monolingual approach (group 2).

**Table 3:** Groups

	Language choice	Number of families
Group 1	Adopted a (more) multilingual approach	8 (5 in England, 3 in Wales)
Group 2	Adopted a (more) monolingual approach	6 (5 in England, 1 in Wales)

### THEME A: Parental Perceptions About the Value of Bilingualism

Parents reached a consensus that bilingualism was valuable for typically-developing children, however their perceptions diverged when it came to the value of bilingualism for children on the autism spectrum.

#### Impact on communication

Parents from group 1 commented on the potential communicative advantages of bilingualism for children on the autism spectrum. When referring to her son’s dual language use, Julie suggested that:

It’s made him have to gauge somebody else’s preferences before he opens his mouth, he’s making those judgements; “do I speak to them in English or Welsh?” So that’s really important, especially when you know, this sort of stereotype of information about autism.

Similarly, Eleanora gave a practical example of how her son distinguishes between languages according to the interlocutor:

He would know to speak English at nursery but he would know that my mother-in-law only speaks Italian, so he would use the language in context, connected to what person he was speaking to.

Magdalena, who opted for a more monolingual approach, also notes a social advantage of bilingualism for her son, namely that having Spanish as well as English offers him another means of communication, and therefore increases his opportunities for social interaction.

#### Cultural Value

Regardless of their ultimate language choices, both groups noted how bilingualism is an intrinsic part of their child’s cultural identity. While Molly commented that she wants her children ‘to be proud of where they’re from’, Baheela’s repetition of ‘our own language’ stressed her sense of ownership of, and identification with, Punjabi:

We mostly use our own language, not the English, we tend to speak Punjabi, our own language.

Nabani raises the potential tension faced by multilingual families with children on the autism spectrum, namely how to maintain their child’s cultural heritage while supporting their linguistic and communicative development (often in the dominant language):

We don’t want to lose their culture. That is kind of like the conflict there. I don’t want him to just speak English because I want him to explore other languages where his roots are and when we do go back to our country I want him to be able to speak in our language as well, where he can communicate confidently.

#### Impact on Cognition

With regard to advantages specific to autism, four parents suggested that being bilingual may offer benefits for their child’s cognition. Roberta described each language as a ‘whole universe’ and suggested that code-switching may increase her child’s cognitive flexibility:

One of the issues with autistic kids is, you know, that they can find it difficult to be flexible in situations, so the fact that he has to switch codes, so with the codes comes a whole universe almost, I think that actually is a good way of practising, you know, flexibility.

In a similar vein, Eleanora highlighted the fact that she had read that bilingual children tended to show advantages in isolating noise and thus hypothesised:

I don’t know whether autistic children need encouragement in that but…I think it might help him later on…like in a big, noisy secondary school, that might help him with isolating, blocking out the noise.

Anna suggested that the increased challenge of switching between languages may encourage her son Dean to ‘keep his mind busy’ and avoid distraction. Only Katherine discussed the possible protective effect of bilingualism on autism, but did not suggest that her decision to raise her son bilingually was based on such a connection.

I think it’s an interesting concept that somebody who is bilingual and has autism, there may be benefits for the autism, in terms of the cognitive flexibility and that kind of thing, but I don’t know whether… that would just be my hunch.

However, some parents expressed apprehension that their children were becoming confused by the presence of two languages:

We thought it’s confusing, he’s getting confused. Which one to pick up. And obviously he stopped and he’s not like other neurotypical children that we see, so better to focus on one. (Hira).

Sometimes he can kind of get mixed up as well, because there’s so much learning in his mind. (Nabani).

Dasia, who at the time of the interview had a grown-up son and daughter, both on the autism spectrum, expressed regrets about raising her children monolingually, but nevertheless commented that her son may have found two languages difficult:

It’s hard to know because there still would have been limitations because of his autism, I don’t know how far he would have gone because of the abstract side of languages, let’s say, the grammar.

Similarly, Magdalena reflected on her own experiences as a language learner and concluded that her son could become distressed when surrounded by the Spanish language, which she describes as an ‘overloading’ experience:

You’re thinking “oh what are they talking about?” and that makes him spiral, you can see he is then overloading because he’s concentrating so much.

### THEME B: Factors Influencing Language Decisions

Language choices were complex for all families in this study. Rather than basing their decisions about language maintenance on their perceptions about bilingualism alone, parents discussed several other key factors in their decision-making, namely: communication with family, advice received, the impact of their child’s autistic presentation on their language development, and the importance of English.

#### Communication with Family

Being able to communicate with family members emerged as the most significant factor for parents choosing to raise their children bilingually. Baheela and Lena comment that bilingualism is a pre-requisite for relationships with extended family members:

When she grows up she’ll be able to speak it and communicate with our parents. (Baheela).

All family is in Poland, you know. He’s going on holiday and he’s going to speak Polish. (Lena).

Two parents who had opted for a more monolingual approach also conceded that bilingualism would aid communication with family outside the UK. Nabani comments that her son ‘would speak Gujarati because all my in-laws are back in Punjab, so when we actually go and visit them he does understand them’. This raises the important distinction between expressive and receptive language for children on the autism spectrum; Nabani had decided that her child understanding the language was perhaps more realistic than him speaking it. For William, Magdalena’s son, speaking Spanish is vital for his relationship with his father, who lives in a Spanish-speaking country. She reports, ‘he has to see his Papá, so it’ll make his life a hell of a lot easier if he can communicate 100%’.

Parents from both groups often based their language choices on what was natural for their family as a whole:

It’s not really based on a theory even though there are theories to say that’s what you should do, it’s just what comes naturally. (Roberta).

But with my children—I never noticed actually—but they understand Hindi and they can speak Hindi, so it’s mostly Hindi because it comes naturally to us at home. (Chandra).

For some families, making a distinct choice between monolingualism and bilingualism was not necessary as their children were diagnosed at age 5 or older and were already bilingual. Nevertheless, Eleanora expressed frustration at the advice she received to speak more English in the home, and provides an important insight into how autism may be missed if practitioners suspect that language delays or limited social interaction are the result of bilingual exposure:

He was in a nursery before and all they were saying was “Oh he just sits by himself and doesn’t talk to anyone, you should speak more English to him at home”. And we were like, “we do. He watches TV in English all the time, our friends don’t speak Italian, he speaks English with them. He’s just not talking to you”.

Parents’ own English proficiency also played a role in language decisions. While the two mothers interviewed with an interpreter were both able to speak some English, it was much easier for them to communicate with their child in their own language, which no doubt played a central role in their language choices.

#### Advice Received

Another key factor in parents’ decision-making was the advice received from practitioners and other family members about bilingualism. Four families (all from group 2) were advised by professionals around the time of diagnosis that one language may be more appropriate for their child:

We were advised to stick to one language because sometimes it can be very confusing jumping from one language to another, and just to keep that consistency as well. (Nabani).

Unfortunately, also we were told at the time when he was diagnosed with autism that it would be best if I spoke one language. (Dasia).

While Nabani intimates that she agrees with the advice, Dasia’s use of ‘unfortunately’ suggests that in hindsight she believes this to be unhelpful advice. Roberta, who chose to raise her son bilingually, said there were family members who were concerned ‘that it would be very confusing if I spoke Italian to the kid and would he be able to speak English?’. Seven families (five from group 1, two from group 2) were given no advice about whether bilingualism would be possible for their child when they were diagnosed with autism, while two parents were advised by practitioners to continue raising their child bilingually. Eleanora describes this process: ‘we did ask the health visitor when he was little and he just said, “No speak both languages and he’ll be fine”, and that’s what we did and his first words were a mixture, some in English, some in Italian’.

#### Feasibility of Bilingualism

Many parents from both groups suggested that their language decisions were based on how feasible bilingualism was for their child. First, parents from group 1 discussed the possibility of their child maintaining both languages. Anna, Katherine and Eleanora commented that children who are deemed to have ‘high-functioning’ autism would be better placed to manage two languages:

If they were like the same ability as Dean, obviously he’s high functioning, I think it’s good for him because his brain is so busy anyway that he can absorb everything. (Anna).

If they had a child like Jamie who was high functioning I would definitely push for it, if the child was non-verbal then it’s tricky isn’t it? (Katherine).

Of course it depends on the type of autism, if it’s high-functioning, that doesn’t sort of come into any of the difficulty. (Eleanora).

Further, Katherine suggested that it is the child’s ability to communicate their basic needs that should ultimately determine whether bilingualism is a possibility:

We’ve been fortunate that for Jamie being bilingual hasn’t had an impact on his ability to communicate his basic needs whereas if he was having difficulties communicating his basic needs then probably we would have gone with just one language.

Conversely, some parents felt that the severity of their own child’s autistic symptoms—that is the extent of their communicative difficulties—rendered bilingualism unfeasible. After seeing her son distressed by her code-switching, Hira opted for a monolingual approach:

Slowly I started working with him at mix-matching and he used to cry and then I said, “it’s fine” and I used to let him cry. “OK, you’re crying, it’s fine” ….and we decided just one language.

Others from group 2 recognised that bilingualism was tenable for some children on the autism spectrum, even if it was not suitable for their own child.

My situation would be that… it’s hard for him. You know and I personally would say don’t push it, but then another kid might be totally different, cos it’s such a spectrum, isn’t it. (Molly).

It's the severity of the spectrum, where you are on the spectrum, how it affects the language. (Magdalena).

#### The Role of English

In certain cases, parents felt that bilingualism was not an achievable goal, concluding that priority should be given to the child’s acquisition of spoken and written English. Davesh, for instance, believed that English should remain the priority for his son. He viewed other languages, including his own native language, as optional:

For me, I prefer him to master English properly in terms of understanding.[…] Basically, because that’s gonna be his primary language for communication. Beyond that, if he wants to learn, I mean I think it’s optional, I think we’d like him to learn Bengali and Arabic.

Similarly, Molly highlighted the importance of English for her son, which was central to her decision to move him from a Welsh-medium school to an English-medium setting:

I feel that if he doesn’t have the English sooner rather than later I might have disadvantaged him.

By contrast, most of the parents in Wales, who were predominantly English-speakers with Welsh-speaking partners, expressed a desire to have been brought up bilingually themselves. This positive view of bilingualism, which was more foregrounded in the experiences of families in Wales than those in England, may have been an important factor in making language choices.

### THEME C: Consequences of Language Choices

#### Changes Over Time

While group 1 opted for a more multilingual approach and group 2 a more monolingual one, it is important to note that these are not dichotomous positions and language practices are, by nature, flexible and in a constant state of flux. As such, discussion of the consequences of parental language choices must be prefaced by the fact that the children in this sample were different ages and this data represents only one point in time; their exposure to different languages inevitably change over time. While Dasia was reflecting back on her children’s development in retrospect, for most of the families, issues of language choices were very much ongoing. This idea of language exposure fluctuating over time is exemplified by Mary’s comment to Anna:

You went through a stage where you thought “right, OK, it’s gonna be better if he just learns one language that we can teach him at home, you know, we can do everything”. But it seems to be working itself out now.

This notion of it ‘working itself out’ was a common thread among parents who opted for a more multilingual approach to raising their child; no parent claimed that it was an easy option, but as the findings of this study demonstrate, many believed their children were now reaping the benefits of being raised bilingually.

Similarly, some families with younger children who had initially opted for a more monolingual approach did not view their decision as fixed. Hira discussed the possibility of introducing Bengali to her son later on, saying ‘when it [English] is built up maybe we can introduce some other things’. Molly commented that although her son would move to an English-medium school from a Welsh-medium one, he would still have some exposure to Welsh. For parents of children with atypical cognitive development, making firm decisions about language maintenance is even more problematic as their developmental trajectories may be more variable or unpredictable than their typically-developing peers. However, one family who had opted for a more multilingual approach discussed their initial concerns.

#### Family Well-Being

Roberta discussed the importance of parental well-being and identity when raising a child on the spectrum, advising:

Absolutely speak to your child in your own language. No question about it. Because you know it can only do good, there is no way it can be bad for the kid. And certainly good for you the parent. Because in everything, you know, I don’t like to be this martyr to my child’s autism. So it’s like, obviously I would do anything but you know let’s not lose myself.

This notion of ‘let’s not lose myself’ underscores the emotional impact of language maintenance in autism not only on children but on parents too. Home languages can play a crucial role in the emotional bond between parent and child, which means that language choices may have significant implications for familial well-being. Other parents from group 2 expressed a sense of guilt or frustration for having opted for a more monolingual approach.

When he goes to see his dad, that inability to be able to express himself must be… it fills his bucket, because he can’t release. He can’t get his frustrations out. So yeah, that’s my fault really, but… it’s life. (Magdalena).

I think had he been given the opportunity to learn the language properly, you know, taking into consideration his autism, I think he would have learned another language. (Dasia).

Magdalena’s use of ‘that’s my fault really’ demonstrates how problematic the choice between monolingualism and bilingualism can be, and the weight of responsibility felt by parents. She went on to justify her decision, suggesting:

I thought he needed to catch up… he was so behind… with the English vocabulary-wise, so we just did some English for a good 2 years, and now… you get stuck in a rut don’t you. (Magdalena).

Again, the use of ‘you get stuck in a rut’ represents the feeling expressed by many parents that the demands of daily life can overshadow language choices, particularly given that parenting a child on the autism spectrum can be more stressful than parenting typically-developing children or children with other developmental conditions (Estes et al. 2013). This sense of culpability persists in Dasia’s account too:

She always makes me feel guilty for not having spoken to her in Arabic. Because she’s very interested in the culture. (Dasia).

In this case, Dasia chose to raise her children monolingually due to the severity of her son’s symptoms. However, this option clearly had implications for the linguistic and cultural identity of her daughter, who was diagnosed with autism at the age of eleven and was more verbal than her brother. The consequences of language choices on the wider family, particularly siblings, is also considered by Roshan, who talks about her non-autistic son:

He completely missed out learning it [Turkish]. They have a one-and-a-half-year difference and he was learning fine so everybody forgot about teaching him any language. He sort of picked up here and there English by himself. He completely missed out because we were focused so much on Zehra. (Roshan).

Roshan continued by describing possible judgement by the wider family that her son did not speak Turkish:

So going to Turkey everyone thinks it’s really weird that he doesn’t… they think we did it on purpose not teaching him Turkish.

#### Education

Parents in Wales had to choose between sending their child to an English-medium or a Welsh-medium school, which had significant consequences for the child’s bilingual development. Molly, for example, was in the process of moving her son to an English-medium school because he was not learning Welsh at the same pace as his peers and therefore could not be assessed in Welsh:

You can’t assess him if he’s not speaking the Welsh and I don’t want to disadvantage him.[…] I just need him to move and I’ll feel more comfortable I think, and it is literally just because of the language barrier.

Anna, whose son was 3 years older than Molly’s, considered the same option for her son but decided it may be too disruptive to change his social setting.

I thought maybe I’ll give him a better chance if he’s in an English school, but then I thought about the social side of it and he wouldn’t have coped with that at all, because all his peers know him and are used to him.

Julie and Katherine both discussed a major challenge for bilingual Welsh-English parents with children with special educational needs; namely, that the lack of Welsh-medium specialist schools means that choosing to raise a child bilingually is not an option in many cases.

We really want him to be educated through the medium of Welsh because we want him to be bilingual and we want him to have all those advantages of being bilingual. But finding a specialist school that will be able to do that is unlikely […].

I think the only thing that is going to be difficult for us, on-going, is whether he is going to be able to stay in a Welsh-speaking school, so in that sense there is not enough Welsh-medium provision for children with additional needs like Jamie. (Katherine).

When we were making that decision about whether to stay in Welsh-medium mainstream or move to specialist education, where it’s such a small pool anyway in specialist education, we weren’t going to find a Welsh-medium specialist school. (Julie).

Only one parent in England mentioned language use at school, which indicates a lack of opportunities for children to use their home language at school: ‘most of the time, even the Polish students speak English amongst each other’ (Lena). Lena’s comments reflect Liu and Evan’s findings (2016) that bilingual students tend to prefer to communicate in the dominant language of the school setting, in this case English, and have less positive associations with their home language when at school.

## Discussion

The aim of the present study was to understand parents’ perceptions about language maintenance in autism and the factors shaping their language choices. This study also sought to shed light on the consequences of these language decisions and draw out commonalities and differences between experiences in England and Wales.

### Perceptions About Bilingualism

As is typical in small-scale IPA research, parents in this study represented a microcosm of diverse experiences of parenting a child on the autism spectrum in a multilingual environment. Eight families opted for a more multilingual approach, while six chose a more monolingual approach. Despite wide variation in the linguistic and social contexts of the participating families, all parents in this study expressed highly positive attitudes towards bilingualism, unlike the mothers consulted in Yu (2009). Parents reported certain benefits of bilingualism specific to autism, most notably enhanced cognitive flexibility and increased social awareness, a result of having to distinguish the linguistic profile of the interlocutor. Participants also highlighted the cultural pertinence of bilingualism for their child, and many felt strongly that speaking the home language was akin to inheriting a cultural identity. However, in keeping with existing research (Hampton et al. 2017; Ijalba 2016; Yu 2016), concerns about confusion, consistency and ‘overloading the brain’ were cited by families from group 2, who had opted for a more monolingual approach.

### Making Language Decisions

The findings of this study demonstrate the complexity of the decision-making process when bilingualism meets autism. Despite their positive attitudes towards bilingualism, parents’ beliefs about the benefits of maintaining bilingualism were sometimes incongruent with the realities of their language practices. Indeed, six of the fourteen families opted for a more monolingual approach to raising their child. Parental perceptions of bilingualism may not therefore be the decisive factor in making decisions about how many and which languages to use. Instead, the parents opting for a more monolingual approach reported that the following factors influenced their decision: communication with extended family; advice received from practitioners; the severity of their child’s autism; and the importance of developing English proficiency. There was a sense in which parents restricted the home language as a means of seeking to promote the child’s development of English, which would best facilitate their ability to communicate.

For families who had decided to maintain bilingualism, the most commonly cited factor was that exposure to the home language would allow children to enjoy relationships with extended family members in a way that monolingualism would not. This finding is consistent with existing research in this area (Hampton et al. 2017; Paradis and Govindarajan 2018; Peña 2016) and suggests that the advice to speak just one language to children from multilingual families may have detrimental effects on their social relationships; this could be particularly problematic given the social challenges that some individuals on the autism spectrum experience. Other factors parents reported in favour of maintaining bilingualism in autism included the fact that their child could feasibly cope with more than one language and practical concerns, such as parents’ own language proficiency. Taken together with the reports from families in group 2 about the challenges of bilingualism, these findings add credence to the recommendation that language choices and practices should be made on a case-by-case basis (Bird et al. 2016b).

### Consequences of Language Choices

With regard to the consequences of parents’ language choices, some parents reported a sense of guilt about their child’s difficulties communicating with extended family members. Others reported regret that their non-autistic child or children had also missed out on becoming bilingual because of their languages choices. In this sense, careful consideration should be given to the emotional well-being of the wider family unit. For example, of those parents who had selected a more multilingual approach, some noted how speaking two languages was the most natural choice for the whole family and enabled the parent to connect emotionally with their children in their own native language. In this vein, many parents believed that bilingualism was not only a possibility for their child, but a conduit for their emotional, social and cultural identities (Jegatheesan 2011; Yu 2013). Mary’s reflection that her grandson’s ability to develop two languages seemed precarious at first but ‘seems to be working itself out now’ showcases the importance of allowing sufficient time for the child to develop as a bilingual.

In Wales, positive attitudes towards Welsh-Medium education were expressed in line with findings from Hodges (2011) and O’Hanlon (2014). However, the consequences of selecting a mainstream or specialist autism school were significant in terms of both the child’s bilingual development and their sense of cultural identity. The conspicuous absence of Welsh-medium specialist provision left some parents in Wales with the dilemma of choosing between English-medium specialist education (and a potential loss of Welsh proficiency) or Welsh-medium mainstream education (without sufficient specialist support). Despite the growing promotion of bilingualism in Wales, Welsh-medium provision for children with additional learning needs is evidently insufficient (Roberts 2017, p. 15).

### Experiences in England and Wales

By drawing on two linguistically different contexts, this study highlighted how different types of bilingualism (i.e. societal vs. individual) may affect parents’ perceptions and choices regarding language maintenance in autism. In Wales, bilingualism is more integrated into the educational system than in England, which may to some extent be reflected in our finding that proportionately more families in Wales opted for a bilingual approach than those in England. Consonant with Hodges’ findings (2011), by sending their children to Welsh-medium schools parents in Wales demonstrated their high regard for bilingualism, despite being mostly monolingual themselves.

Parents in England, by contrast, who were all bilingual, were more hesitant about the possibility of bilingualism for their child. For most of the parents in England, English was their second or third language, so they may have considered proficiency in English to be a high priority for their children. Although it would be ill-advised to make generalisations about such a small sample, it is possible that families’ wider linguistic setting has some influence on their perceptions about language maintenance. Another important difference between the two settings was that, unlike in England, no family in Wales received advice about bilingualism; given the growing bilingualism in Wales, more support needs to be available so that parents can make research-informed decisions not only about the language used in the home, but also the type of education their child receives.

## Conclusions

### Strengths and Limitations

This study offered unique insights into perceptions, factors and consequences that relate to maintaining bilingualism in autism. The broad range of language backgrounds of the children and their families in this study usefully reflects the diverse linguistic make-up of the UK. To our knowledge, this was the first study of its kind to consider the interaction of autism and bilingualism in Wales. Crucially, our findings that no parents in Wales were advised about language maintenance despite its bilingual education system and that parents in Wales faced a difficult choice between school type (mainstream or specialist) and language of instruction (English or Welsh) have important implications for policy makers in Wales.

The use of interpretative phenomenological analysis as a methodological framework in this study helped to illuminate the ‘insider perspectives’ (Reid et al. 2005) of all participants, giving voice to a variety of experiences and providing in-depth insight into the unique challenges they faced. By giving participants the choice of interview location, access to interpretation and an opportunity to expand on and clarify their comments through member-checking, their authentic voices could emerge.

More information about the children’s language proficiency and exposure—acquired through a language background questionnaire—may have enriched the contextual understanding of our analyses in this study. Additionally, gathering detailed information about parents’ own language backgrounds as well as other demographic information such as age, country of birth and education may have provided more significant trends between groups regarding perceptions about bilingualism and would benefit future research. A further limitation of this study was the over-representation of mothers; understanding fathers’ perceptions of maintaining bilingualism in autism may have yielded different results and requires further examination, particularly as this group are so under-represented in autism research (Martins et al. 2013). Finally, given changes in language practices and attitudes over time, a longitudinal design may have provided more accurate results regarding the consequences of language decisions in autism, and should be explored in future research.

### Implications

As this study has demonstrated, parents’ perceptions about the value of bilingualism are only one factor of many when deciding whether to maintain bilingualism with their child on the autism spectrum. The findings suggest, however, that adopting a more multilingual approach may positively impact upon children’s communication with both immediate and extended family and increase parental well-being. By contrast, a more monolingual approach may engender negative consequences for wider family life such as parental guilt or the prevention of siblings’ bilingual development. Accordingly, greater attention must be paid to the advice given to multilingual parents of children on the autism spectrum, especially in view of the lack of advice received by parents in this study.

Providing training and guidance for practitioners (speech and language therapists, paediatricians, teachers, and health visitors) on the complexity of language choices would be a useful step in ensuring that families make appropriate decisions. Along with Beauchamp and MacLeod (2017) and Lim et al. (2018), we call for greater support for families who wish to adopt a bilingual approach to raising their child with a developmental condition like autism and a greater awareness among practitioners about the possible negative consequences of advising a purely monolingual approach. Our findings suggest that in Wales there is a need for more specialist provision through the medium of Welsh so that parents are not having to choose between sending their children to specialist autism schools or raising them as bilingual Welsh-English speakers. Drawing on the Welsh context, this finding may also be relevant to a vast number of settings where educational systems offer provision in two or more official languages.

Despite the growing body of evidence to suggest that bilingualism is not, broadly speaking, detrimental to children on the autism spectrum, the findings of this study confirm that there will be some children for whom a single language approach may be most appropriate. In this vein, our findings corroborate Baker’s view that ‘neither the pole of single-language nor multilingual immersion should prevail unilaterally’ (2013, p. 533), but instead assessing and addressing the needs of the individual should be prioritised.

### Future Research

This study highlights the need for more research into the effects of bilingual exposure on individuals on the autism spectrum as well as how autistic traits affect children from culturally and linguistically diverse backgrounds (Lim et al. 2018). Along with Hampton et al. (2017), we recommend that further research explores how language choices influence the well-being of children on the autism spectrum and their families. Given that the severity of autistic symptoms and the child’s ability to communicate their basic needs emerged as key factors in making language decisions, more critical attention should be afforded to the impact of bilingual exposure on non-verbal children on the autism spectrum, as well as those with limited speech. Future research should reflect the perspectives of all stake-holders—parents, educators, speech and language therapists, health professionals and the children themselves—in helping families to make well-considered choices about which, and how many, languages to use.

## Footnotes

1 In Kenny et al.’s study, the terms ‘on the autism spectrum’ and ‘autistic’ were preferred by the autistic community while professionals were much more reluctant to use the term ‘autistic’, and preferred ‘person with autism’. However, as their study reports the views of adults, it may be injudicious to assume the terminological preferences of children with an autism diagnosis without consulting them. As such the current study adopts the term ‘on the autism spectrum’, rather than ‘autistic’ or ‘with autism’.
